# The Principal Forms of Insanity in Relation to Treatment

**Published:** 1846-07

**Authors:** 


BRITISH AND FOREIGN

### MEDICAL REVIEW,

FOR JULY, 1846.

### PART FIRST.

### Analytical and Critical Reviews.

### Art. I.

*Die Hauptformen der Seelenstörungen in ihren Beziehungen zur Heilkunde.* Von Dr. Maximilian Jacobi.

*The principal Forms of Insanity in relation to Treatment.* By Dr. Maximilian Jacobi.—*Leipzig,* 1844. 8vo, pp. 822.

Of the continental writers of the present day on the subject of insanity, few, if any, have acquired a higher reputation than Dr. Maximilian Jacobi. Although perhaps his opportunities for observation have not quite equalled in extent those afforded by the larger lunatic hospitals in England and France, yet so much accurate discrimination has been exercised in the still numerous cases which have fallen under his notice, and so much practical knowledge has been displayed even in some of his earlier works, that he may, without doubt, be looked upon as one of the highest authorities in all questions relating to disorders of the mind.

The lunatic asylum of which he is the director is at Siegburg, situated about five miles from the town and university of Bonn, on the eastern bank of the Rhine. It is devoted to the accommodation of insane patients from the Prussian Rhenish provinces. Jacobi's first work was entitled, 'Collections for the Treatment of Disorders of the Mind' (Sammlungen für die Heilkunde der Gemüthskrankheiten), and consists of two volumes. The first, published in 1822, contains an account of "The Retreat" near York, a translation of Mr. Samuel Tuke's work; and the second division, a translation of various articles respecting insanity, contributed by Esquirol to the 'Dictionnaire des Sciences Médicales,' never collected in the French language until published shortly before his death by the author, in his last great work, entitled 'Traité des Maladies Mentales,' &c. The subject of the second volume is 'Psychical Phenomena, and their relation to the system in a healthy and diseased state.' Here we have some account of his physiological theory concerning the relation of the brain and of the system in general to the mind, which, in the course of his other works, he more fully develops. In 1830 Jacobi published a work, called 'Observations concerning the Pathology and Treatment of Diseases connected with Insanity' (Beobachtungen über die Pathologie und Therapie der mit Irreseyn verbundenen Krankheiten), the most important and valuable part of which consists of a series of very interesting cases, related with great minuteness. In the former part of this book, after some introductory remarks, he brings forward a theory, in which, however, he is far from being peculiar, viz. that there is no disease of the mind existing as such, but that insanity exists solely as the consequence of disease, either functional or organic, in some part of the bodily system. The third section is taken up with critical remarks upon the works of Esquirol, Georget, and Guislain; and in the fourth we have some imperfect statistical account of the insane in the Prussian Rhenish provinces. The number of lunatics is computed to be 1 in 1000 inhabitants. The remaining two thirds of the work are devoted to the description of twenty-seven cases of insanity of various forms, interspersed with the conversations and writings of the patients themselves, both in prose and poetry.

In 1838 the first volume of the 'Journal for the Diagnosis and Cure of disordered Conditions of the Mind' (Zeitschrift für die Beurtheilung und Heilung der krankhaften Seelenzustände) was edited by Jacobi and Nasse of Bonn, and published at Berlin, containing a series of essays upon subjects connected with insanity—cases, prophylactic treatment, a description of English asylums, &c., by the editors, and by Drs. Flemming, Jesse, and Zellen, who are directors of lunatic establishments in Germany. The English asylums, his visits to which are described in this Journal by Jacobi, are Hanwell, St. Luke's, New Bedlam, Maidstone, Oxford, Stafford, Lancaster, Liverpool, Wakefield, and the Retreat near York. The construction of these houses is a subject to which considerable attention has been paid by the author. Besides the translation of Mr. Tuke's description of the Retreat, inserted in his 'Sammlungen,' Jacobi afterwards wrote a separate work, laying down principles and directions for the erection of suitable buildings for a hospital for the insane, with a particular notice of the establishment at Siegburg, of which he is the director. This treatise has been translated from the original language and published with introductory remarks by Mr. Samuel Tuke. It is unquestionably the best work extant on the construction of lunatic asylums.

The present work of Dr. Jacobi will, when complete, consist of three volumes, only one of which is as yet published. It is divided into five sections:—1st, a series of cases; 2d, an account of the pathological phenomena which occur in diseased conditions connected with mania; 3d, the causes of mental disorders having the character of mania; 4th, the course of diseases connected with mania; and 5th, the treatment of mental diseases. In his introductory remarks we find the following classification of the various forms of insanity.

I. Alienation of the appetites or propensities, comprising a state of exaltation, being *mania*, and a state of depression, being *melancholia*.

II. Alienation of the intellectual powers, subdivided into *illusion* and *dementia*.

III. To these two classes we have a third added, which also comprehends two divisions, viz. *delirium* and *idiotcy*, forming altogether six species of insanity, which he proposes to describe. This first volume is devoted to the consideration of mania alone. The second will contain melancholia and illusions: the third, the remaining forms of insanity.

In its general plan the volume before us bears considerable resemblance to the celebrated work of Louis on phthisis. The inferences and observations in the second part are founded upon a strict examination of several accurately recorded cases, which are given in detail in the first part.

The cases are separated into three groups, which are thus arranged:

1st. Cases of mania (Tobsucht) without illusion (Wahnsinn), with but little disturbance of the intellectual powers.

2d. Cases of mania with delirium or incoherence (Bewirrtheit) without illusion.

3d. Cases of mania with illusion, being subdivided into two sets—mania in one and illusion in the other class being the predominant feature.

All the cases, with the exception of some which are called "Outlines," are drawn up with that accuracy and attention to minute details in their histories which is the result of the admirable system of clinical instruction prevalent in most German medical schools.

Before we pass on to notice the second section of this volume, in which are contained the author's remarks and inferences from the fifty cases which he reports, it will be worth while to extract one from each group, to assist us in understanding the succeeding parts of the work. These—and the others are like them—will probably startle and provoke our readers, from their astounding lengthiness; but we prefer giving a few of these cases in all their fulness, to abridging many of them, as it is on the fidelity of his individual portraits that the author must be judged. Unquestionably many of the details are unnecessarily minute, and a careful revision of the MS. might have saved the reader from not a little unnecessary diffuseness and repetition. The cases, however, as they are here delineated, will afford valuable studies to the practitioner; and, we doubt not that their perusal is capable of supplying important hints to most of our clinical physicians that have to deal with insane patients.

In the division of cases exemplifying mania without illusion, the first is interesting, as it gives us an example of the disease in a low stage of its development, and serves to illustrate the accuracy with which the commencement of the disease is investigated. We give it in full:

"CASE.—J. R., a man of strong bodily constitution, had just passed his 37th year when he was brought, in September 1839, to the asylum. He was born of healthy parents, in good circumstances, who were remarkable for their intelligence and good dispositions, and no other case of insanity was known in the family. The patient also in his early life, although an unruly boy, had shown proof of excellent abilities, and was on that account intended by his parents for some literary occupation. After having been sent to school he soon became disinclined to this mode of life, and discovered a strong inclination for farming, was fond of horses and dogs, and delighted in hunting. As his father possessed considerable landed property, and his brothers had devoted themselves to other pursuits, it became easy for him to follow his inclination, and he had acquired so much skill that the whole of his paternal estate was left under his care, and improved yearly by his exertions. Until his twentieth year his health had been apparently good. In that year he suffered an attack of insanity, which continued for six weeks, and this recurred in a similar way seven times, with intervals of two or three years. The progress of the attack was every time as follows: At first the patient became for a short period dull and depressed, and this state was followed by a continually increasing excitement, which in its highest degree only manifested itself in an excessive eagerness in following his ordinary pursuits. His love of horses and dogs, and of hunting, was very much increased, as was also his activity in business. He was in the greatest degree enterprising and full of projects, and restless in everything which he took in hand, performing with his own hands the hardest agricultural labour from morning till night; showing, moreover, an immoderate degree of self-confidence with a tendency to bursts of anger, and an evident weakness of judgment at the same time, avoiding the society of his family, and conducting himself towards them in an unusually repulsive and contemptuous manner. At these periods he usually spent sleepless nights, without any decrease in his overstrained activity during the day. His appetite was in a like manner increased, and whilst he was generally (except during the attacks of violence) very moderate in the use of spirituous drinks, he now showed a great inclination for them, without, however, becoming intoxicated. During the attacks no decided disorder of the intellectual powers ever occurred, although transient illusions were remarked. Altogether, his insanity especially manifested itself in an exaltation of all the active powers of the mind, and, above all, in an impulse to vehement expressions of the will and propensities. When, after the lapse of four or five weeks, this condition had reached the highest pitch, then the excitement speedily subsided, and he returned to his usual physical state only after an interval of a few days of depression corresponding to that with which the attack ordinarily commenced. The patient himself, even during the continuance of his attacks, had a consciousness of some disordered condition, and was always depressed and anxious after they had passed off, and several times expressed a wish to be brought to this institution to undergo treatment; and at last gave it as his decided wish, as the attack before the last had been more violent and obstinate than those which had previously occurred, that upon the recurrence of the disease, even if at the time it should be against his will, his friends should bring him hither. This wish was acted upon. But as his friends were convinced that during the attack he would not submit to the measure, it was performed with the assistance of an officer of the police; at which he was very discontented, pretending that he would have gone willingly; nor could he for a long time forgive his brothers for their share in this proceeding; yet he evinced no particular dislike to the establishment, and was willing to follow the directions there laid down for his treatment. Moreover, upon his arrival the attack had already nearly reached its highest point, and the period of decline soon began when after the usual depression he regained his healthy state. The question now was, what plan was to be adopted in order to secure the patient from future attacks of a like nature?

"It was impossible to doubt that the tendency to these morbid phenomena which developed themselves during the attacks, as well as the attacks themselves, had their origin in a disordered exaltation or irregular excitement of the brain and whole nervous system. But from what source did this change arise, and how was it maintained? We found after pressing inquiries that this individual, so powerful and leading so active a life, had been from his boyhood up to his present adult age excessively addicted to masturbation. Being continually tormented by his conscience, and even full of anxious thoughts lest the attacks of disorder of his mind should be caused by this vice; always making new resolutions to renounce it, and after a short period of determination, during which he kept his resolutions, breaking through them; always driven to doubts from his weakness, and at the same time incapable of summoning strength for a continued renunciation; always feeling himself struggling to avoid this frightful precipice, and driven to it again, his mind suffered no less than his body, and the disordered condition of his brain was no doubt the result.

"The patient, who was now convalescent from his mental disorder, willingly acquiesced in a plan of treatment which was to emancipate him from an evil which always threatened him, and which was so much dreaded. A spare diet, avoiding soup and meats difficult of digestion, especially in the evening, a cool regimen, continued active employment in the open air, sleeping in a "camisole" near the attendant, general cold baths with cold douches on the perineum, the internal use of iodine, and lastly, means calculated to strengthen his moral power, and revive his courage to contend against the propensity which had overcome him, were the chief means adopted to subdue so destructive and deeply-rooted an evil. And these means, with the cooperation of the patient, did not disappoint our expectations. He preserved himself free from vice during several months. But as in this complete and continued state of psychical good health he could no longer be kept in the establishment, and as he himself wished to return to his business, where he could not conveniently be spared, he left Siegburg at the end of May, cheerful and with the most earnest intentions to behave himself continently. No further account has been received of him." (pp. 1 et seq.)

The case is not without interest, but it scarcely amounts to what in this country would be called mania. The propensity to drink showed itself during the attack, and was not habitual with the patient; whereas the other vicious habit is said to have been the cause of the disorder. In many cases when these depraved propensities exist, it is extremely difficult to ascertain whether we are to look upon them in the light of cause or effect.

To illustrate the second group, or those of mania with delirium or incoherence with illusion, we extract Case 20:

"J. M., a countryman, 52 years of age, well made, lively and excitable, was brought to this establishment in July. He had been engaged in the expeditions of the French into Spain and Russia from the year 1806, and had been addicted to great excess in drinking and debauchery; had been repeatedly infected with syphilis; and after narrowly escaping being drowned in the Beresina, lost the ends of his toes from the cold. At the end of the war he married, and lived quietly at home. He was, at the time of his arrival at the asylum, suffering from an illusion with a considerable degree of excitement, believing himself to be in the possession of great riches and of a high station, and busying himself with continually changing plans for employing his property, &c. Yet his ideas of wealth never became perfectly boundless (which we have frequently observed to be a symptom of incurable organic degeneration of the brain), and he recovered in a few weeks; so that at the beginning of September he was able to return to his home. In the following year, about the season of his first attack, after continued hot weather, the disorder of the mind recurred, having now the character of mania with illusion. In the first days of July he complained of violent beating and noise in the head, but until the 6th he showed no sign of insanity. On this day, after he had drunk an excessive quantity of brandy, and eaten about three pounds [!] of meat, the disorder of the mind showed itself with such violence that it was found necessary after two days to bring him back to the asylum.

"The patient was in good bodily health; the conjunctivæ were reddened; the look changeable, lively and gay, his eye bright, with a fixed, contracted pupil, his chest moderately expanded; a closer examination detected slight dilatation of the bronchial tubes, and hypertrophy of the left ventricle. The viscera of the abdomen showed no sign that should lead us to suspect any particular disease; his appetite was very good, tongue moist and almost clean, and the bowels acted freely. The powers of voluntary motion and the functions of the organs of sense were unimpaired. Common sensation was also unaffected, with the exception of a transient pain in the head. Sleep was almost entirely wanting. The phenomena of mental disorder were the following: The patient continually talked of military affairs, at one time with loud shouts giving the word of command, swearing, and calling to generals and officers known to him in the service, &c.; but during this violent maniacal excitement no single idea was continually present; the pictures in his mind appeared rather to rise as involuntary reminiscences than as the effect of an illusion. He was moreover dirty, indecent, shameless, and often very violent, his violence being directed rather against inanimate objects than his neighbours, towards whom he at the same time behaved himself in a friendly and even jocose manner; as, for instance, once when he managed to free himself from his strait-waistcoat, he quickly put it upon an idiotic patient who was near him, and led him to the attendant. There was a continual change from paroxysm to remission, neither of which often extended beyond ten or twelve hours, the former prevailing generally throughout the night. In the progress of the disease for the first two months the paroxysms continued to increase in length and intensity, and then became somewhat shorter and less severe, although he had occasional attacks of the most violent nature. The pulse was very changeable in frequency and quality from the commencement, the difference not being caused by more intense maniacal excitement or remission, nor did it correspond with those different states. In point of frequency it was seldom under 80 beats, often being 92-96, but only occasionally reaching 100, and this generally during the period of decided remission, there being at the same time very little force in the artery. The tension and impulse were much less in the radials than in the carotids, which generally beat very strongly, whilst at the same time all the veins of the neck were turgid and pulsating, owing without doubt to the existing disease of the heart. For two days only, during the exhibition of the infusion of digitalis, the pulse sank to 66, and in one day, when the patient was unusually quiet, to 60: but on the next day, under the continued use of the same medicine, and whilst the patient remained in the same tranquil state, it rose to 90, and in the following day, under great maniacal excitement, it fell again to 68, 60. The secretion of saliva was very copious, the bowels were daily moved, his tongue was clean and moist, and his appetite was always very good, except during a few days, in which it appeared to be lessened by the use of digitalis.

"The patient was treated upon the principle that his brain and nervous system, from the first very irritable, after having undergone, together with his whole system, destructive influences of various kinds—such as the effects of climate, repeated attacks of syphilis, and the means of their cure—were now suffering from the consequences of a repeated and continued over-excitement, with its still persistent high degree of irritability, and also from irritation of the nervous ganglia of the abdomen, which were in a similarly disordered state, and from irregularities in the circulation of the blood consequent upon disease of the heart. This treatment (as was the case almost without exception in all the soldiers who had taken part in the French, Russian, and Spanish campaigns, and in whom insanity had appeared in this form) was entirely ineffectual. Towards the end of September a general affection of the system appeared, followed by œdema, ascites, and hydrothorax. All means which were adopted proved of no service in averting the continual progress of the disease, and he died upon the 2d of January—that is, just half a year after the second attack of insanity.

"At the commencement of the more severe bodily diseases all the signs of mania disappeared, and the patient became more sensible; so that during the last weeks of his life he might be considered as tolerably free from insanity, although his intellect was somewhat weakened. It was observed that he was sometimes delirious in the night, but this had no evident connexion with his insane condition." (pp. 172 et seq.)

A minute account is given of the morbid appearances on dissection. We will notice only those of the head:

"The scalp was œdematous and full of blood upon the left side; skull cap of moderate thickness and density, saturated with blood upon the left side. The dura mater firmly adherent to the skull, thickened at the falx major, and adherent to the arachnoid and pia mater, in which some serum was infiltrated. The substance of the brain was in the upper part of the hemispheres of moderate consistency, very much softened at the fornix, the third ventricle, and the posterior cornua of the lateral ventricles. Some fluid was found in the left lateral ventricle. The commissura mollis did not exist. The corpora quadrigemina appeared somewhat roughened. Traces of softening were found in the fourth ventricle." (Ibid.)

Cases of mania with illusion are divided into two classes: those in which the maniacal symptoms predominate, and those in which illusion is the most prominent character. To illustrate the first let us extract the 44th case, or rather a part of it; for it is so very long and minute that we fear no English reader would peruse it in full:

"CASE.—J. H. R., a young agriculturist, was brought to the asylum, labouring under mania, three times in the course of seven years, and recovered completely each time. The first attack took place in August, 1828, when he was 23 years of age. He was a well-made young man, whose external appearance seemed to promise continued good health. In his childhood he had shown himself at least of moderate intellect, and, as far as could be discovered by inquiry, no case of insanity had occurred in his family. Three years ago, however, he had been ill for three weeks with a nervous fever, attended by obstinate delirium; and it appeared that from that time a diseased excitement of the brain existed: his temper during the same time had been very irritable. After his recovery from the fever he had been considered to be in perfect health, and had since devoted himself continually to his employments with great activity. In June, 1828, in the middle of the day, as he was returning home in company with many other young men, who with him had been subjects of the conscription, he suddenly became violently excited, loaded his companions with unfounded reproaches, and behaved himself altogether like a man who had lost his reason. During the five following days he remained in the same condition, some hours, however, passing in which he was perfectly sensible and quiet. On the sixth day he lay in bed, said that he was ill, and had continued illusion. His face was red and swollen, and he had great thirst and disinclination to take food. The disease during the following days became more serious, with continued fever, muttering, delirium, and a dark brown coat upon the tongue. An experienced physician was called in and upon the administration of the remedies he so quickly improved that upon the fourteenth day he could leave his bed, and appeared quite sensible, complaining only of lassitude and want of appetite. After the lapse of eight days his face became again flushed, his conversation was irrational, and violent attacks of mania followed, returning after a certain remission, but preceded always by a considerable flushing of the face. He became quite ungovernable, so that it was necessary to keep him constantly under restraint, and in this state he was brought to the asylum on the 28th of August. He continued some days in the same condition, his nights being quiet. The pulse was 80-85, his face flushed, temperature of the body heightened, especially in the palms of the hands, his appetite good, and digestion unimpaired. After the first part of September the paroxysms became gradually weaker; he was quiet and obedient, and began to employ himself, but he was still mad to a certain extent. In the second half of the month he became again much more violent and troublesome, so that he was obliged to be strictly guarded; the pulse showing no great tension, and amounting in frequency scarcely to 75, afterwards to 66, whilst the temperature of the skin was natural, the face being at the same time redder than usual. These exacerbations soon passed off again, and in October the state of his mind became quite natural, and remained so; and after the lapse of a few months of trial he left the asylum at the end of March, 1829, perfectly well. No critical secretion was perceived upon his convalescence, but he was decidedly stouter and more healthy in appearance than before the commencement of the disease.

"R. remained well until the end of February, 1833. At this time he began to suffer from cold feet, heavy sleep, and constipation. On the evening of the 22d he was found kneeling in prayer in the open street; and upon returning home he complained of a noise in the head, and expressed a fear that he was again about to be ill. Five days afterwards he had another attack of mania, in which the face was very red; he cried out violently, passed his evacuations involuntarily, and talked much of God and the saints. After the abstraction of some blood he became quiet, and continued so for the five following days with alternations of mania, and from that time till the middle of March he was tranquil, when he returned to his occupations; but upon the 16th the disease returned with such violence that he was hurried back to Siegburg upon the 19th.

"On his arrival his skin was dry and of a yellowish-gray colour, his head hot, and extremities cold; he said that he was free from headache. Digestion was natural, the function of the lungs was properly performed. The first eight days were spent in the most violent state of mental excitement, without his taking any fluid whatever; he slept during the night generally undisturbed. Occasionally illusion manifested itself, and he declared that he was St. John, and that he would be so styled; this, however, was not very permanent. The disordered condition, as in the former attack, was considered to be a greatly increased irritability of the brain, combined with weakness, and accompanied by an excited state of the vascular system, by which the irritation of the brain was produced. The following observations were made respecting the further course of treatment of this attack:

"On the 20th of March the patient was flushed, his head hot, he was covered with perspiration, his feet cold, pulse full, 100, 105 in a minute, that in the carotids not being unusually strong; he was turbulent and noisy, but upon the application of cold to the scalp, and mustard baths to the feet, he became tranquil after a few hours; the temperature of the scalp was lowered, the pulse was slower, small, and weak, the respiration quiet and gentle, and he passed a quiet night. On the 23d he was again excited and greatly disturbed, he spat in every direction around him, and licked up the saliva of the other patients. He was confined on the chair, cupped upon the back of the neck, cold applications were made to the head, and he was given dilute sulphuric acid with water. His condition changed continually from moderate excitement to tranquillity until the beginning of May, when the flushed and heated state of his head accompanied another attack of violent mania, and continued in a greater or less degree throughout the summer. Digitalis was now given in the form of powder, from $\frac{1}{2}$ to $1\frac{1}{2}$ gr. four times daily for two months, and at the same time shower-baths were used. The pulse was very changeable during the use of this remedy, 95, 100, 140, 110, 120, 78, but at last, on the 6th of July, it sunk to 43 beats, and on this day the patient was particularly violent and noisy. When most frequent the pulse was altered in quality, but not depressed; when least frequent, it was smaller and softer. Digitalis was now omitted, and the treatment was confined entirely to an unstimulating diet, and a corresponding moral regimen. The psychical condition remained unaltered, so that the maniacal excitement continued through August, the pulse differing at different times, not exceeding 70 beats, sometimes being as low as 48. In September the pulse was more steadily low, and the patient, whose illusion with the mania had remained unaltered, although the former showed no influence upon the latter, began to be more quiet and manageable; his convalescence then advanced so rapidly for a few weeks that at the end of the month he could be considered well, and no unnatural condition of any organ could be discovered, except the slowness of the pulse, which was 55, and which by very slow degrees returned to the natural state, which in this individual was 75-78. He was retained during the winter in the asylum, to preserve him if possible from a relapse, and from the use of intoxicating liquors, which were so dangerous to him, and in the beginning of March he was dismissed.

"Twelve months afterwards he was taken ill in a similar way. On the 15th of March (until which day he had remained in perfect health) he complained that "his skin had adhered to his body," and a few days afterwards he considered himself a rich gentleman, and his brothers his servants. He became then suddenly maniacal, and tore and destroyed everything he could lay hold of. He was confined, became quiet, and slept during the night, showing no trace of mania for fourteen days, although he still considered himself a rich man, and would not trouble himself by doing any work. On the 30th of March he had another attack of mania, which compelled his friends to bring him again to the asylum, where he arrived on the 2d of April. Upon his arrival he displayed great maniacal excitement, cried, spoke abusively, tearing his clothes, menacing us with blows, &c. He again had the illusion that he was St. John. After a few days the excitement gradually abated, returning again speedily; and this change continued with short intervals for six months, whilst the illusion was always present in the same way, although it exerted no evident influence over the paroxysms of mania. The attacks during this illness were slighter than in the earlier ones, so that they only showed themselves in excitement, and not as real outbreaks of fury. The temperature of the head and the colour of the face were much heightened, and the patient appeared always more confused after he had warmed himself with exercise. Digestion was unimpaired, his tongue clean, appetite good, and the bowels were in a natural state. As our former view of the cause of the disease might be looked upon as well founded, the treatment in this attack remained on the whole the same. At first tartar emetic was given with nitre, and from the end of June to the beginning of September digitalis, in the form of acetum digitalis, in increasing doses afterwards in conjunction with aqua amygdal. amar. At one time twenty leeches were applied to the arms, and afterwards twelve cupping-glasses to the back of the neck, and for a long time daily for several hours cold applications to the head. The frequency of the pulse varied this time also under the use of digitalis, but not to so great an extent, as it never exceeded 100, and only once sank below 70. We not unfrequently observed great tension of the arteries with fulness, but without any real strength; it preserved in the carotids its natural relation to that in the radials.

"The maniacal condition, which had continued in the above-mentioned form during the whole of the summer, began to yield gradually to this treatment at the end of August, and disappeared entirely in September.

"From this time R. appeared to be in a perfect state of physical and mental good health; but he wished to remain longer in the asylum for fear of another relapse, and therefore applied to be made attendant—a request which was readily complied with, as, during his convalesence, he had shown himself to be very sensible, attentive, and careful. He remained in this situation without a return of his illness for nearly three years, and then went away to marry and keep house for himself." (p. 273.)

The last case (48) which we extract will illustrate the second subdivision of the third order, viz., mania connected with illusion, where the illusion predominates:

"Case.—N. J., 40 years of age, had served as grenadier under Napoleon for ten years, and had received many wounds. He afterwards had some small civil appointment, and married a woman much younger than himself, with whom he lived peaceably, and had eight children. But now his income was insufficient for his increasing family, and he was tempted by want to appropriate a small sum that had been intrusted to him; and not being able to replace it, became melancholy, and suffered from extreme anxiety and restlessness. Not long afterwards, in the month of May, he began occasionally to act in a foolish manner, to talk unconnectedly, and to scream out in the night. Instead of the sleeplessness which had lasted to the present time, he suffered from exceeding drowsiness, a feeling of depression, and at the same time an enormous voraciousness. This condition having lasted till the middle of the summer, a continually increasing excitement was observed, and at last, about the middle of September, his wife was obliged to call in medical assistance. The patient now manifested an unusual degree of impetuosity, rashness, and hastiness in all his movements. He himself complained of extraordinary disquiet, oppression of the chest, faintness, headache, and unrefreshing sleep, much disturbed by dreams, he said he had no peace or repose at home, and even his old wounds in his head and arms began to smart. The pulse was at the same time full and hard, his countenance unusually red and swollen, with spasmodic twitching of the eyelids, the conjunctivæ traversed by enlarged vessels, a wild look, voracious appetite, bowels confined, tongue but little coated, skin dry, and its temperature natural. According to the account given by his wife, he had slept but little during the last six weeks, was quarrelsome, and did not as usual amuse himself with his children. After two days he became much worse; and being quite sleepless he leaped out of bed in the night, seized his wife by the hair, dragged her about the room, and attempted to strangle her. At the same time his conversation was indecent, he abused his children, was dirty in his person, suffering from the highest degree of excitement of the genital system, with the greatest shamelessness, whilst his mind was filled with illusions respecting the conduct of his wife, and the persecutions to which he was subjected.

"After venesection (the blood exhibiting the inflammatory coat), the patient became for a moment quiet, and said that the oppression on his chest was lessened. On the next day, however, the morbid excitement of the mental faculties was much increased; he was more shameless and filthy, shouted, abused, and tried to injure his friends, and at the same time he was under an illusion that all men wished him ill, and tried to rob him of his bread, that he was unable to support his family, &c.

"He was brought to the asylum a fortnight afterwards, in the beginning of October. He was a tall, powerful man, with a scar, three or four inches in length, over the occipital bone, resulting by a sabre cut received at the battle of Austerlitz; the scalp was very moveable, its temperature not too high, the eye very unsteady, pulse moderate, the lungs and heart healthy, bowels regular, uninterrupted sleep during the night, and no present manifestation of mania.

"His mind was, notwithstanding, much disturbed by many illusions and discordant ideas, the influence of the disordered condition manifesting itself in his judgment, especially in the false interpretation of all the acts and words of those around him, to whom he imputed the most hostile intentions, the object of which he either was or feared to become. He was full of hatred and ill-will, mischievous and lying, and always endeavoured to present an appearance of justice and truth; he avoided all acts of violence, although sometimes a trace of furious excitement was perceived, and the maniacal element of the disease, which at first was very prominent, had now disappeared, and cowardly meanness appeared in its stead. The longer this state continued the stronger was the disorder of his imagination, and the more his behaviour was perverted. Thus he endeavoured, among other things, to remove the keys from the doors, secretly to destroy the walls and plaster, to throw away any tools or clothes which he could secrete, took the lining out of his breeches, in order to have an opportunity of complaining that they gave him no drawers, fastened his gloves beneath his coat, and cursed the attendant for exposing his hands to the cold during the winter frost, &c. He persevered in the idea of the injustice, oppression, and neglect which he daily had to undergo, and every prudent regulation which was acted upon on account of his violence merely confirmed his illusion. He seemed, however, to have occasional lucid intervals, in which he acknowledged that his conduct was perverse, and promised not to repeat it. These intermissions seldom lasted to the next day, and were generally only the signs of a more excessive alienation. In the summer months he was somewhat better, although he still dissembled and was inclined to abuse and slander. The pulse (which was scarcely affected for two days consecutively by digitalis taken for a long time) was for the most part 80-85, frequently sharp, but without strength and fulness; the bowels were regular, appetite good, tongue clean, and sleep quiet.

"Upon the 24th of December the patient suddenly manifested great excitement, with hastiness in his movements and incoherence in his ideas, and the following day he was perfectly maniacal. This condition, now appearing for the first time since his arrival at Siegburg, remained for eleven months stationary, accompanied with an ungovernable propensity to destruction, and ended only with the life of the patient; remission for a few days having been occasionally observed. He frequently spent sleepless nights in continual motion, screaming so violently that he was hoarse for several days afterwards; frequently, however, his nights were quiet, and his fury commenced by day. His unconnected raving bore the stamp of the most extreme impudence and indecency, and he passed his evacuations in his bed and clothes. He ground his teeth in a terrible manner, and tried to bite and tear everything that he could reach; he tore to pieces several strait-waistcoats during the day, rendering it necessary to put on a wire mask, and in the night, when his hands and arms were confined, he tore to pieces his bed and bed-linen with his feet. The pulse was changeable, 85, 90, 78, soft, and otherwise quite natural in the carotids and radials during the maniacal attacks. No increase of temperature was observed during the course of the disease.

"The plan of treatment which was pursued was quite ineffectual. Before the beginning of spring a general state of ill health, from the influence of the disordered condition of the nervous system upon the organs of physical life, came on and increased during the summer; œdema of the feet, abscesses in the extremities, cough, with copious expectoration of yellow mucus, diarrhœa, emaciation, and general weakness. He died on the 6th of November, twenty-five months after his reception into the asylum.

"On dissection a considerable quantity of serous fluid was found in the left side of the chest, but scarcely two ounces in the right, whilst about five ounces were found in the pericardium, which, after exposure to the air for a quarter of an hour, coagulated into a gelatinous mass. Considerable hypertrophy of the left ventricle, the aorta was dilated at its arch, and its internal coat, as well as that of the innominate artery, was softened with atheromatous deposits of the size of a pea in different parts. The abdomen contained some fluid; the liver was large, pale, and bloodless, and so was the spleen. In the cranium the sinuses and veins of the brain were full of black blood, the arachnoid opaque, without any thickening. The consistence of the brain was less than usual in all parts, especially in the more deeply-seated regions. The hemispheres were firmly united together above the corpus callosum for two-thirds of their length, so that it was requisite to divide them with a scalpel, in order to arrive at the great commissure. The two lateral and the third ventricles were filled with fluid." (p. 300.)

The preceding cases will suffice to display the style of Jacobi's observations, and they are worthy of note in another point of view. The frequent bleedings and the use of depressing means which were adopted in the majority of the cases, are part of a method of treatment which in this country finds at present but few advocates.

The second division of the work gives an account of the pathological phenomena which occur in diseased states connected with mania, and consists of conclusions drawn from a comparison of the fifty cases which are contained in the first division, and which are for this purpose arranged in four tables.

I. The first table gives the name, age, sex, constitution, and temperament of the patient, the number and character of the pulsations at the wrist and in the carotids, with the state of the functions of the heart, lungs, and skin during the attacks of mania, and during the remissions or the period of convalescence.

With regard to the frequency of the pulse no general inference can be drawn, although the number is accurately noted in every case; the chief peculiarity being the quickness with which variations occurred both in the number and quality of the pulsations in the carotids and radials. In certain attacks of mania the pulse mounted to 100-130, whereas in other attacks in the same individual it was even less frequent than during the remission; nor do the observations which were made in order to ascertain if any variation occurred at different times of the day give any positive result, beyond the variations caused by accidental circumstances, as, for instance, by the upright or horizontal position, &c. The cases in which the pulse of the carotid corresponded in quality to that of the wrist bore the relation of 7 to 1 over those in which the pulsation was stronger in the neck.

The action of the heart corresponds with the pulse, but by means of the stethoscope in several instances symptoms of hypertrophy were discovered during the remission, which during convalescence entirely disappeared. A similar remark is made respecting the state of the lungs, viz. that the physical signs of tubercular deposit, as mucous râle, dulness on percussion, and considerable resonance of the voice were frequently present during an attack of mania, but vanished on recovery taking place. Esquirol remarked the frequency of organic disease of the heart and lungs in cases of insanity, and Foville says that five out of six of the bodies of lunatics which he examined displayed organic disease of the heart, generally hypertrophy, but the observation of their transient nature is of great importance and peculiar to Jacobi. He accounts for these anomalies by imagining that the nutrition of the parts has been influenced and altered by the affection of the brain and nervous system. In half the cases there was no change in the temperature of the skin; but in the rest it was more frequently raised than lowered; and in the half of those in which no alteration existed in this respect, the colour of the face was notwithstanding evidently heightened.

The observations which were made in order to draw up these tables are additional to the general description of the cases, some specimens of which we have extracted, and furnish what may have been thought wanting in them, namely, a close account of the state of the different organs and their functions; and here Jacobi finds fault with other authors and observers, and remarks, that by scarcely any of them has any real attention been paid to these important particulars; that even Esquirol, in forty-five cases which are scattered throughout his great work, has given the state of the pulse only in one instance. But it is not fair to conclude from the fact that Esquirol did not publish in systematic tables like those of Jacobi the results of his experience, that his observations are not close inductions from facts actually noted down by him. Esquirol was through his life a most careful observer of facts, which he recorded with remarkable accuracy, and afterwards arranged and systematised with a rare sagacity and insight into their relative importance. His general observations are the deductions which he himself made from a multitude of individual facts; Jacobi's method of laying before his readers in a tabular form a collection of phenomena, from which inferences are to be drawn, has a more imposing effect and taxes less the faith of his readers as to the accuracy of the inductions, but we doubt whether better and sounder opinions will be formed by many readers on the data submitted to them, than those which Esquirol offered in a tone of confidence which he felt that he had a right to assume.

It follows from the facts stated in these tables that no condition of the pulse either with respect to its frequency, quality, or the relation between the pulse at the wrists and in the neck, will afford any assistance to diagnosis in cases of mania. This negative result does not prove that it is unnecessary to examine the pulse in lunatics; it is highly important with regard to variations occurring at any particular time in any individual case, but it shows that no general rule can be laid down, and that descriptions of mania giving as a diagnostic mark a quick hard pulse, &c., are either taken from one single individual, or are merely the preconceived notions of the author.

Another important fact which Jacobi upholds in opposition to the opinion of many writers is, that a febrile state, or pyrexia, is not the usual concomitant of a maniacal attack; and as he gives us the numbers, the accuracy of which we have no reason to doubt, he is certainly more entitled to credit than those who only mention the opposite fact in a general way. Out of 222 cases seen in the establishment at Siegburg, only 22 or 23 presented symptoms of fever, many of which were hectic, and other forms of pyrexia, having no necessary connexion with the mania.

According to Dr. Burrows, who considers that an abnormal condition of the pulse is closely connected with the psychical state of maniacs, there is a remarkable difference observable in many cases between the number of beats in the radial artery and in the carotids, and even in this respect the two carotids not unfrequently differ from one another in the same individual. This observation has been confirmed, or rather repeated, by other writers. One case is mentioned by Burrows, where the pulse at the wrist was 90; that in the carotids 115, 120. Jacobi evidently treats this case with considerable suspicion, and takes some pains to contravene the opinion of Dr. Burrows, and adds, "that of 1200 patients who were repeatedly the subjects of observation by him and the assistant physicians of the establishment, *in no one instance* had the number of the pulse in the radial and carotid arteries, or in the two radials, differed to the amount of one single beat." It would certainly be very strange if facts should turn out so contrary to all our physiological notions respecting the circulation as to induce us to believe that the pulse is not produced by the contraction of the heart, or that any cause between the heart and the wrist can have the effect of lessening the number of pulsations to such an extent. It is perfectly intelligible how there can be a difference in the quality of the pulse, even between the two carotids, from a diseased condition of one hemisphere of the brain, as we find in inflammation of the hand that the radial artery beats much more fully and strongly than upon the opposite arm, with which, however, it corresponds in point of number.

Jacobi remarks, with regard to this difference in the quality of the pulse of the two sides, that when it is constantly present the case is almost always incurable.

Compression of the carotid arteries in cases of mania was first imagined and tried by Dr. Parry: it has been employed by Dr. Burrows and also by Jacobi at Siegburg. Burrows says, that in recent cases of mania the violence of the symptoms was lessened by the practice. Jacobi observes on this point:

"With respect to the result of this operation (compression of the carotids), which has been so frequently performed in our asylum, we must first of all remark that the occurrence of phenomena following it varied exceedingly in different individuals, and that in some they were scarcely to be detected. They were as follows: a feeling of burning heat which spread suddenly over the head and neck down to the chest, or, when the compression was merely upon one side, over the corresponding side of the head and neck, with a feeling of heat within the head in many cases. The face becomes darker, with frequently the production of a vivid colour, an extremely painful sensation of compression of the chest, a feeling of tension, weight, and pain in the head, giddiness, staggering, sleepiness; actual sudden sleep, with stertorous breathing; in many incipient syncope, with uncertainty in the use of the lower extremities, stumbling, and in some cases they suddenly fall down with entire insensibility, but speedily recover. One of these patients fell before me at the moment when both carotids were compressed, as if struck down by a blow, but came to himself in a few moments, and complained of a headache; in another the syncope was not so complete, continued for some time; and a third said, when he recovered from the state of insensibility, looking wildly around him, that he had suddenly fallen asleep, and could not collect himself upon waking, so as to remember where he was." (p. 383.)

We here have a collection of symptoms in all probability arising from violent pressure, not only upon the carotid artery, but also upon the veins and nerves of the neck, and we are not surprised at finding that Jacobi considered the plan of compression of no practical value in treatment, although he adds that, when carefully employed, the appearance or absence of any of the above-mentioned results of the proceeding may afford considerable assistance in the diagnosis of the case.

II. The second table refers to the state—1st, of the upper part of the alimentary canal, teeth, gums, appetite, thirst, &c.; 2d, of the lower part of the digestive organs; 3d, the function of the kidneys and condition of the urine; 4th, of the skin; and lastly, of the functions of the entire apparatus of nutritive or organic life. The results of these observations are simply remarkable for the very little departure from the usual condition of health which they exhibit. The secretion of saliva was frequently increased and the appetite was often excessive; notwithstanding this latter fact, however, the nutrition was in the generality of cases in a very low state, but there was no constant disorder in the digestive system. On this point the experience of Dr. Jacobi is contrary to that of all English and French practical writers who have observed that the function of secretion and the state of the mucous membrane of the alimentary canal in maniacs is very much disordered, so much as to cause a peculiar fetor which has been noticed by very many observers. In the third section of his 'Observations,' upon the pathology and cure of mental disorders, Jacobi endeavoured to disprove an observation of Esquirol, who remarks that there is a smell peculiar to the insane, proceeding partly from the skin and partly from the breath, although in the present work he passes it over unnoticed. He accounts for the increased desire for food as well as for the abundant secretion of the salivary glands by the somewhat unsatisfactory expression of an alienated condition of the functions of the nervous system, and he devotes a considerable portion of this section to extracts from other writers, which he compares, or rather contrasts, with his own observations. He has looked through the writings of other authors, in which descriptions of cases have been given, and has found that out of 130 which have been described in various works, the state of the tongue is mentioned in only 13, that of the bowels in 25, and the condition of the salivary organs and secretion in only one, while no writer gives a description of mania without mentioning particularly the condition of the various organs. The tendency of these remarks would lead us to look upon such general accounts of the diseases, when they differ from his own, even though narrated by Esquirol himself, with a certain degree of suspicion.

The fulness of these tabular statements has certainly the advantage of bearing an appearance of authenticity and weight; but it must be evident that the observation of the practitioner may be diverted by the great number of particulars which he is called upon to notice from directing itself to symptoms which, to a mind not so engaged, would present themselves as essential and characteristic.

III. The third chapter and table is drawn up under four heads, and gives—1st, a statement of the age and temperament, the form of the skeleton, development of the muscles, colour of the hair and iris; 2d, the state of the organs of sense, and sleep; 3d, that of the brain and nervous system, exclusive of the higher psychical powers; and 4th, that of the organs of generation.

The muscular system was, in the generality of these cases, moderately developed, and the degree of bodily strength was in relation with it. Esquirol, Ideler, Pinel, Neumann, Rush, and, in fact, almost every writer upon the subject describe the muscular power as excessive, enabling the patients to break their chains, &c., whereas Jacobi asserts from his own observations that no increase whatever in the strength takes place, and that a single attendant is generally able to overcome a patient during an attack of maniacal excitement, so as to put him under restraint.

The senses were slightly disordered in 12 out of 50 cases, in the generality of whom the abnormal condition consisted in an increased sensibility of the ear and eye, a dislike to loud sounds, and a slight degree of intolerance of light: common sensation was in an altered state in many cases; and in a large proportion sleep during the night was almost entirely absent.

Paralysis and other disorders of the brain and nervous system were not very frequent. In most cases the brain was the first part of the nervous system which was affected, while in some the disease was said to have commenced in the sympathetic—in others simultaneously in both sets of nerves.

Among the male patients but very few exhibited any signs of inordinate excitement of the generative organs, and among the females the only remarkable fact was the frequency with which the catamenial discharge was found wanting, returning in some when the mania became confirmed—in others as a commencement of convalescence.

IV. The fourth table gives an account of the morbid psychical phenomena in maniacs; in the appetites, moral feeling, and exercise of the will; in intellect, imagination, association of ideas and judgment, as well as the state of the mental faculties during the remission.

After giving an account of the relative numbers in which the various phenomena presented themselves, a repetition of which would be of no interest to our readers, the author remarks:

"From this table it is seen that a general violent psychical excitement, hastiness, and mental irritability, with tendency to break out into fury and rage, exists almost always in mania, united with an inclination to act violently towards others, a love of destruction, inattention to the rules of cleanliness, sometimes also with a complete alteration in the natural disposition of the individual; whilst, on the contrary, morbid excitement of the genital organs, rudeness of manners and lewdness, which are not natural to the patient, are only decidedly seen in the minority.

He describes as the source of their behaviour an ungovernable change of the appetites, sometimes displaying itself but slightly, but generally breaking out into excess of malicious, insolent merriment, occasionally preluding a state of depression and anxiety. The exact subjects or material which furnishes them with ideas during the period of maniacal paroxysm are traced by Jacobi to three sources:—1st, objects which present themselves to the cognizance of the senses, as we so frequently see in the delirium of typhus; 2d, pictures of past events called up in the memory; and, 3d, single isolated words, phrases, or numbers which the patient remembers and repeats without cessation, and frequently with the most violent gestures. And here we must recollect that out of 228 cases of mania which the author notices, only 20 showed any illusion to such an extent as to become an element in their ideas during the period of excitement.

Dr. Jacobi considers that this form of madness differs from a merely excited state of the mind produced by illusions, and that during the attack itself certain causes (momente) act upon the mind, and influence the maniacal language and gestures, whereas most authors consider that a disorderly and confused state of the ideas, or even an illusion, is the exciting cause of the paroxysms. Esquirol says that the multiplicity of the ideas, and the rapidity with which they are received, the faulty association, and the deception of the senses, and want of attention, lead astray the judgment of the patient, and by over-exciting and over-straining his passions drive him on to violent and dangerous extremes.

The third division of the work, 'On the causes of mental disorders which have the character of mania,' is subdivided into four chapters:—1st. General influences, circumstances of climate and weather, condition of the country, food, family, morals, political state of the people. 2d. Influence of sex, age, bodily constitution, temperament, intellectual abilities, religion, occupation. 3d. Hereditary and congenital causes. 4th. Acquired, remote, and exciting causes.

The statistical or numerical results presented to us in this part of the volume are based on too few data to be admitted as general conclusions. We have access to data much more extensive, which we hope to lay before our readers on another occasion; we shall therefore, for the present, pass over the principal results of this portion of our author's work. We must, however, find room for a brief notice of his peculiar views respecting the nature and causes of insanity.

Most authors consider mental or moral causes as of more frequent operation than physical in producing diseases of the mind; but, according to Jacobi, those who think thus formed their opinions not upon the accurate observation of facts, or, at any rate, they only observe those occurrences which are immediately antecedent to the maniacal attack. This remark of the author respecting the psychical causes of mania in some measure involves the consideration of a view peculiar to him. He thinks, for instance, that the brain is not (as most observers, from its structure and from pathological facts, are led to conclude) the only organ of all psychical actions, but that the bones, ligaments, muscles, in fact every part of the system, has an equal share in producing the phenomena which are usually referred to the mind! This is in a few words the substance of his theory, to which we alluded in the commencement of the present article, when speaking of his former works, and of which the following is an abridged account, taken partly from his 'Sammlungen,' and partly from the work now under consideration.

After giving quotations from various authors who consider the brain as exclusively the organ of the mind, he says that if we will examine the facts which tend to elucidate the relation of psychical phenomena to the entire organism of the human body, we shall necessarily be led to conclude that besides the brain and the nerves, the whole system, as well as the individual organs, are in immediate relation to the original production, and to the various forms of these phenomena. In regard to the bony fabric, for example, he says that, from the improved manner of examining such subjects, the time will come when the physiologist will contemplate the os sacrum quite as fully as the os frontis, if he wishes to determine what had been the degree of mental endowment of the individual, and "when the form of the skull being given, it will be no proper trial of skill if he is called upon to describe the mental powers of a Philoctetes or an Ulysses." How often, he continues, do we see peculiarities in the skin and its appendages, hair of a certain colour or strength, and peculiar cutaneous secretion, temperature, &c., connected with some particular condition of the mental powers? while varieties in the state of the mental faculties are continually observed upon the recession or reappearance of cutaneous eruptions, &c. The vascular system, he says, is on all hands allowed to be closely allied to mental phenomena; a man with a large development of the arterial system is active, hopeful, sanguine, and enterprising; and a contrary condition of the vascular system is found in men disinclined to active exertion, and slow in judgment and resolution. The organs of voice have similar indications, and in like manner the stomach and alimentary canal; for instance, different kinds of food produce different effects. "Some men are incapable of deep thought in the morning before they have taken coffee; some require half, some an entire bottle of wine to make them sociable." Similar facts are brought forward respecting the muscular and other systems.

Holding, as he does, such views as these, it is perfectly intelligible how Jacobi can admit that diseases of any part of the system do not act upon the mind and produce insanity through the medium of the brain. Being not less than the brain parts of the organism connecting the mind to the body, the imperfection of the instrument in any instance prevents the possibility of a natural or healthy action of the mental faculties. We may connect with this idea of Dr. Jacobi the fact of his leaving out of his list of causes one which is of frequent occurrence, and which is always enumerated in other works upon the subject, viz. injuries of the head. Although there is a certain amount of truth in his remark concerning the occasional occurrence of insanity in cases which do not manifest cerebral disease, and are dependent on the disorder of other organs, his view is utterly untenable as a true theory of insanity; and certainly he has been able to establish the truth of no such thing in any part of his writings.

We find nothing in the sections on treatment which would interest our readers. It is evident that the system of non-restraint, as carried out in this country with such admirable results, is imperfectly known and practised at Siegburg.

In conclusion, we must say that although the present volume contains much that is valuable, and merits a place in the library of every physician who treats insane patients, or studies insanity, it will add much less to the previous stock of our knowledge, and to the widely-spread reputation of its author, than we had anticipated before perusing it.

---

